# The complete plastid genome of a medicinal tree *Lindera chienii* Cheng 1934 (Lauraceae: Laureae)

**DOI:** 10.1080/23802359.2022.2093675

**Published:** 2022-07-04

**Authors:** Chao Liu, Huanhuan Chen, Jian Cai, Lihong Han

**Affiliations:** College of Biological Resource and Food Engineering, Yunnan Engineering Research Center of Fruit Wine, Qujing Normal University, Qujing, China

**Keywords:** *Lindera*, chloroplast, phylogeny

## Abstract

*Lindera chienii* Cheng 1934 is an important medicine plant. The first complete plastid genome sequence of *L. chienii* was assembled and analyzed in this study. The plastid genome is 152,744 bp in length with a GC content of 39.15%, contains a large single-copy region of 93,767 bp and a small single-copy region of 18,843 bp, which were separated by a pair of inverted repeat regions of 20,067 bp. A total of 128 genes were detected in the plastid genome, including eight ribosomal RNA genes, 36 transfer RNA genes, and 81 protein-coding genes. The phylogenomic analysis based on plastid genomes supports the close relationships among *Lindera chienii*, *L. megaphylla* and *Litsea acutivena*.

*Lindera chienii* Cheng 1934 (Lauraceae), a dominant evergreen shrub, is an important medicinal plant distributed in the Provinces of Zhejiang and Anhui in China (http://www.iplant.cn/foc). The essential oil from leaves of *Lindera* shows a stronger inhibition than amoxicillin against *Staphylococcus aureus* and *Candida albicans* (Wei et al. 2016). *Lindera* belonging to the core Lauraceae, is often confused with genus *Litsea* and *Laurus* (Liao et al. 2018; Tian et al. 2019; Song et al. 2020; Liu et al. 2020; Liu et al. 2022). For a better understanding of the relationships of *L. chienii* and other Laureae species, we assembled and analyzed the complete plastid genome of *L. chienii* for the first time.

Fresh leaf samples of *L. chienii* were collected from Nanjing Zhongshan Botanical Garden (Jiangsu, China; Long. 118°50′3.19″ E, Lat. 32°03′9.67″ N, 45 m). The voucher was deposited at the Biodiversity Research Group of Xishuangbanna Tropical Botanical Garden (Accession Number: XTBG-BRG-SY36085, Song Yu, songyu@xtbg.ac.cn). Total genomic DNA were extracted with a modified CTAB method (Doyle and Dickson 1987). Genome was sequenced on the Illumina HiSeq 2000 platform at BGI-Shenzhen. About 1.7 Gb pair-end (150 bp) raw reads were obtained. The plastid genome of *L. chienii* was assembled and annotated using GetOrganelle pipe-line (Jin et al. 2020) and GeSeq (https://chlorobox.mpimp-golm.mpg.de/geseq.html) with *Lindera glauca* (MG581443) served as the reference.

The complete plastid genome of *L. chienii* was 152,744 bp in length. The plastid genome possessed a typical quadripartite structure, consisting of a large single-copy (LSC) region (93,767 bp), a small single-copy (SSC) region (18,843 bp), and two inverted repeat (IRa and IRb) regions (20,067 bp). A total of 128 genes were found in the plastid, including 36 transfer RNA (tRNA) genes, eight ribosomal RNA (rRNA) genes and 81 protein-coding genes. The GC content of the complete plastid genome was 39.1%, and those of LSC, SSC, and IR regions were 37.94, 33.90 and 44.43%, respectively.

In order to investigate the phylogenetic relationship between *L. chienii* and related species in Laureae, the complete plastid genome sequences of *L. chienii* and other 49 taxa in Laureae were aligned by MAFFT v7.450 (Katoh et al. 2019). Maximum likelihood (ML) phylogenetic analyses were performed by the IQ-TREE v2.1.1 (Minh et al. 2020) with 1000 bootstrap replicates, and the best model TIM + F+R2 was selected based on IQ-TREE (Figure 1). The result showed that *Lindera* species grouped into four clades. *L. chienii* was located in the same clade with two *Laurus*, five *Litsea*, and seven other *Lindera* species. *L. chienii* is closed related to *L. megaphylla* and *Litsea acutivena* with 100% bootstrap value.

**Figure 1.**
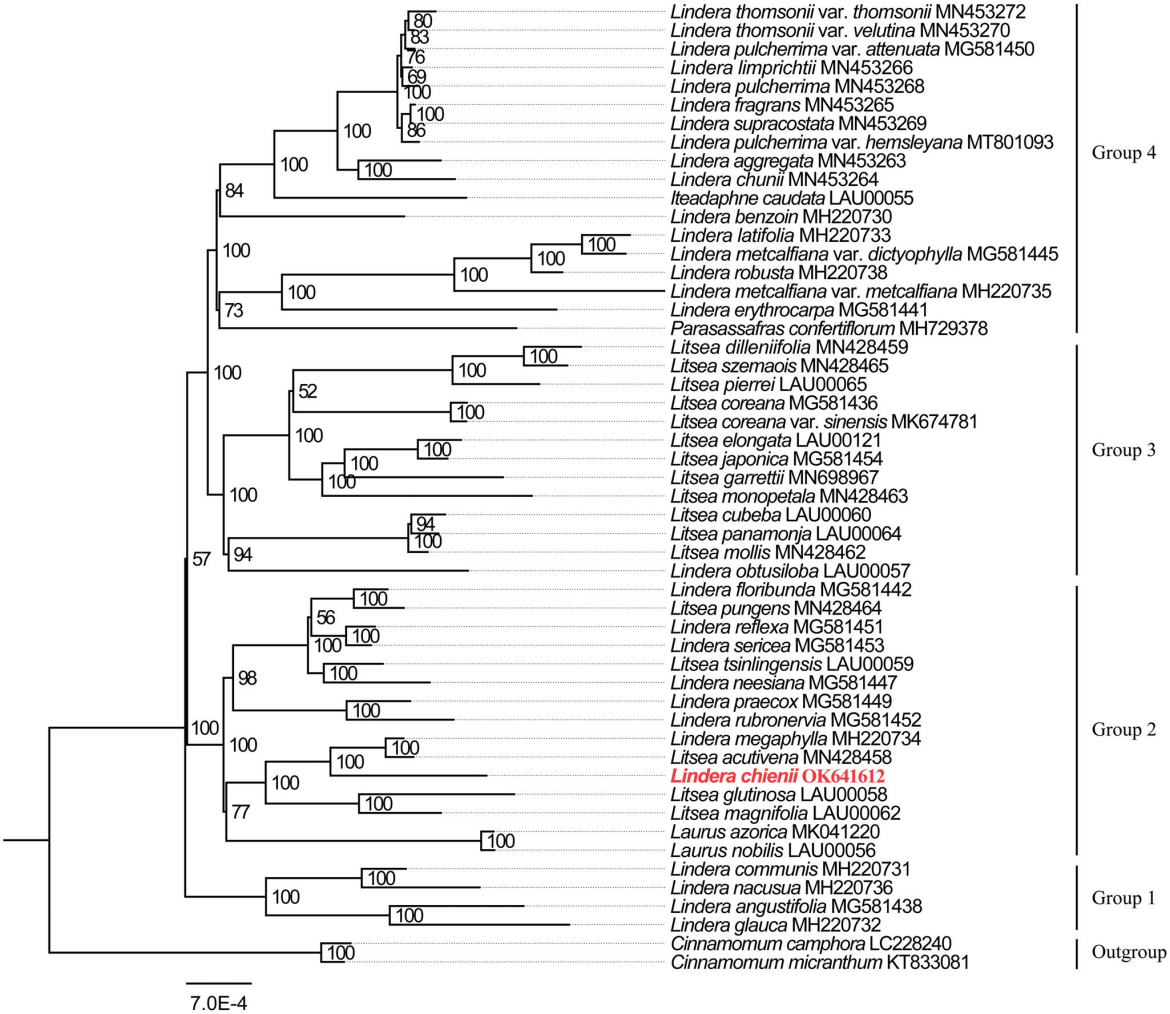
The maximum-likelihood phylogenetic tree constructed with plastid genomes of Laureae.

## Data Availability

The genome sequence data that support the findings of this study are openly available in GenBank of NCBI at [https://www.ncbi.nlm.nih.gov] (https://www.ncbi.nlm.nih.gov/) under the accession no. OK641612. The associated BioProject, SRA, and Bio-Sample numbers are PRJNA778332, SRR16841626, and SAMN22942607 respectively.
